# Citric acid treatment reduces decay and maintains the postharvest quality of peach (*Prunus persica* L.) fruit

**DOI:** 10.1002/fsn3.1219

**Published:** 2019-09-27

**Authors:** Can Yang, Tao Chen, Borui Shen, Shuxia Sun, Haiyan Song, Dong Chen, Wanpeng Xi

**Affiliations:** ^1^ College of Horticulture and Landscape Architecture Southwest University Chongqing China; ^2^ Horticulture Research Institute Sichuan Academy of Agricultural Sciences Chengdu PR China; ^3^ Key Laboratory of Horticulture Science for Southern Mountainous Regions Ministry of Education Chongqing PR China

**Keywords:** citric acid, organic acids, peach, polyphenols, sugar, volatile compounds

## Abstract

Peaches are easily perishable fruit, and their quality is quickly lost after harvest. In this study, “Hujingmilu” peach (*Prunus persica* L.) fruit was treated with citric acid (CA) and stored at 20°C for 15 days. Fruit decay and quality were evaluated during the storage period. Compared with the control, CA treatment did not inhibit climacteric ethylene release, but CA was significantly effective at maintaining firmness, inhibiting decay, and preventing a decrease in titration acid (TA). CA treatment inhibited the increase in total soluble solids (TSS), sucrose, and fructose in the first week after fruit harvest, but then their content was significantly higher in CA‐treated fruits than that in the control group. The decrease in malic acid and citric acid was significantly prevented by CA treatment. During storage, the concentrations of C6 volatile compounds decreased rapidly whereas lactones sharply increased, and the concentrations of δ‐decalactone, γ‐decalactone, and γ‐dodecalactone were found to be significantly high in CA fruits compared with the control after the eighth day of storage (*p* < .05). Similarly, higher contents of chlorogenic acid, neochlorogenic acid, catechin, and L‐epicatechin were maintained in fruits treated with CA during the same storage period (*p* < .05). Our findings suggest that treatment with 10 g/L citric acid can reduce postharvest decay and effectively maintain the texture, flavor, and nutrition quality of peach fruit.

## 1 INTRODUCTION

Peach (*Prunus persica* L.) is one of the most commercially important Rosaceae trees cultivated widely in the world, with total production of 19 million tons approximately (FAO, http://www.fao.org/faostat/en/#data/QC). As a typical climacteric fruit, peaches will undergo undesirable changes in texture, flavor, and nutrition quality after harvest, resulting in rapid softening, water and flavor loss, flesh browning, and rotting, and ultimately lose their commercial value (Lurie & Crisosto, 2005). Thus, peaches are extremely perishable after harvest; the shelf life of peach fruit is generally only 5–7 days at ambient temperature. It is estimated that the postharvest loss of peach fruit accounts for 30% ‐ 40% of total production (Hodges, Buzby, & Bennett, 2011). These problems will cause serious economic losses and become one of the key issues that restrict the sustainable development of the peach industry.

Postharvest treatments can retard aging, delay the deterioration, and extend the shelf life of fruits. Currently, chemical treatment, cryopreservation, and controlled atmosphere storage methods are extensively used to prolong the storage life of peach fruit. However, chemical treatments leave residues on fruits and low‐temperature storage causes chilling injuries (CIs) to the fruit and decrease the specific ester contents and flavor quality of peach fruit with increased storage time (Ortiz, Echeverria, Graell, & Lara, 2009; Xi et al., 2012). Although controlled atmosphere storage has effect on reducing postharvest fruit rot and maintaining quality, long‐term storage is expensive (Ortiz et al., 2009).

Citric acid is often considered safe and, as an organic acid, can be used as a food additive (Sommers, Fan, Handel, & Sokorai, 2003). The previous study has shown that citric acid not only inhibits the growth of bacteria and fungi in fruits and vegetables but also induces the improvement of disease resistance in vegetables (Patrignani, Siroli, Serrazanetti, Gardini, & Lanciotti, 2015). It can also prevent browning and fruit disease by reducing the respiration of postharvest fruits (Pilizota & Sapers, 2004) and is a good additive to improve the acidity and flavor of foods, thereby improving the quality of preservation and storage and preventing food spoilage. The use of citric acid for fresh‐cut water chestnut can maintain its food quality and extend its shelf life (Jiang, Pen, & Li, 2004). Citric acid treatment can slow down the decrease in the soluble sugars and titratable acidity and is beneficial to maintain the fruit quality of Chinese jujube fruits during storage (Zhao et al., 2009). Thus, citric acid treatment is potentially an ideal fruit preservation approach. However, the effect of citric acid treatment on perishable fruits such as peaches is currently unknown.

In the present study, we aimed to investigate the effect of citric acid treatment on fruit decay incidence, fruit firmness, and ethylene release from peach fruit, with emphasis on developing a novel strategy for improving the postharvest storability of important phytochemical‐related fruit flavor and nutrition quality, including soluble sugars, organic acids, aroma volatiles, and the main phenolics. Our findings suggest that citric acid can provide an effective approach to extend the shelf life and maintain the postharvest quality of peach fruit.

## 2 MATERIALS AND METHODS

### 2.1 Fruit material

“Hujingmilu” peaches were collected from the Chengdu Academy of Agricultural Sciences in Longquanyi District, Sichuan Province, China. Fifteen trees of the same age and similar growth state were marked for collecting experimental fruits. Fruits with uniform color, shape, and size and without mechanical and pest damage were selected at commercial maturation stage and then transported to the laboratory on the same day.

### 2.2 Citric acid treatment and storage conditions

A total of 30 fruits were used to determine the initial fruit quality parameters, with ten fruits representing one replicate and three biological replicates being used. Another 1,260 fruits were divided into two groups, one group for CA treatment and the other as the control. The CA group fruits were sprayed evenly with 1.5 L (10 g/L) citric acid. After drying naturally, the fruits were placed and sealed into nine cold storage box with multiple vents. After being closed for 24 hr, the vents were opened and ventilated. The control group was treated with pure water according to the same procedures. All cold storage boxes were placed at 20°C ± 0.5°C, and the relative humidity was maintained at 85% ± 1%. A total of 90 fruits were sampled at 2, 4, 6, 8, 10, 12, and 14 days after treatments, respectively. Thirty fruits represented one replicate, and three replicates were used for each sampling.

### 2.3 Determination of decay incidence, total soluble solids, titratable acidity, and firmness

Before determining the fruit quality parameters, thirty fruits as one replicate was used to investigate the decay incidence and three biological replicates were used. The decay incidence was calculated according to the following formula: decay incidence (%) = (number of decayed fruits/ total number of fruits) × 100%.

The total soluble solid (TSS) and titratable acidity (TA) were measured by using a B32T Brix Meter digital saccharimeter (Atago Co., Tokyo, Japan). The pressed juices were directly dropped on the refractometer to measure the TSS, and the results were expressed as %. The juices were diluted 100‐fold with pure water to determine the TA content using the refractometer, and the content was expressed as %. Three biological replicates were used for each sample.

Firmness was measured with a GY‐4 type hardness tester. A 1‐mm‐thick peel was cut on both sides of the fruit with a knife. Then, an 8‐mm‐diameter head of the GY‐4 hardness tester was used to measure the firmness of the fruit at 1 mm. Three biological replicates were used for each sample.

### 2.4 Determination of ethylene production

Ethylene production was determined according to our previous work (Xi et al., 2014). Three peach fruits were weighed and placed in a container sealed with a rubber stopper and maintained at a constant temperature of 20°C for 2 hr. The 1‐milliliter gas sample at the top of the vessel was then aspirated using a microsyringe and injected into the GC unit for testing. Ethylene production was measured by an Agilent 6890N GC unit equipped with a DB‐WAX (0.32 mm, 30 m, 0.25 μm, J&W Scientific, Folsom CA, USA) capillary column and a hydrogen flame ionization detector (FID). The amount of ethylene release was expressed as the nL/g. Three biological replicates were used for each sample.

### 2.5 Determination of soluble sugars and organic acids

The extraction and identification of soluble sugars and organic acid were performed as described in our previous study (Xi et al., 2014). A total of 2 g of frozen fruit powder were completely homogenized in 5.0 ml of ethanol (80%). The solution was maintained for 2 min, and the supernatant was used to extract the soluble sugar and organic acid using a hot water bath for 20 min at 35°C. The extraction was repeated three times. After centrifugation at 10733 *g* for 10 min at 4°C, the supernatant was combined and increased to 25 ml with ethanol (80%). One mL of the above volumetric solution was dried at room temperature using a nitrogen blower (SE812J). The residue was then dissolved completely with 0.5 ml of double distilled water and filtered through a 13‐mm water membrane with a pore size of 0.22 μm, and the filtrate was used for the analysis of soluble sugars and organic acids.

The soluble sugars and organic acids were separated by HPLC (Waters 2,695, USA) equipped with a 0.5 μm NH_2_ (4.6 × 250 mm) column (Torrance Technologies Inc., USA) and a Waters C18 (4.6 × 250 mm) column (Waters, USA), respectively. A refractive index detector (Waters 242, USA) and a Waters 2,996 diode array detector was used for the identification of soluble sugars and organic acids at a wavelength of 210 nm, respectively.

A standard curve was prepared by setting the concentration of the standards to cover a range of sample concentrations. The soluble sugars and organic acids were quantified according to the standard curve, and their concentrations were expressed as mg/g FW. Three biological replicates were used for each sample.

### 2.6 Determination of volatile compounds production

Aroma volatiles were determined using our previous method (Xi et al., 2014). Three grams of frozen powder were placed in a 20‐mL headspace extraction bottle; 5 ml of saturated sodium chloride solution was added, and 10 μL of ethyl nonanoate solution (5.77 mg/ mL) was added as an internal standard. After vortexing for 10 s, the solution was incubated 30 min at 40°C, after which the volatiles were extracted using a solid‐phase microextraction (SPME) needle coating with a 50/30 µM divinyl benzene/carboxen/polydimethylsiloxane (DVB/CAR/PDMS) fiber (Supelco, Bellefonte, USA).

After equilibration for 30 min, the extract was disported for 4 min and then detected by GC–MS with a Rt × 21MS capillary column (30 m × 0.25 mm × 0.25 μm). The GC oven temperature started at 40°C, was increased to 200°C at 3°C/min and then to 250°C at 5°C/min, where it was maintained for 5 min. Helium was used as the carrier gas, and the flow rate was 1.2 ml/min. The temperature of inlet, transfer line, and detector was 250°C, 250°C, and 300°C, respectively. In electron ionization mode, the electron energy was set to 70 eV, the scanning range was 60–600 m/z, and the ion source temperature was 200°C.

The compounds were tentatively identified by comparing their mass spectra with the data system library (NIST 98), linear retention indices (LRI) reported in the literature, and EI mass spectra data from authentic compounds. The concentrations were calculated using the standard curve method, with the results presented in µg/kg FW. Three biological replicates were used for each sample.

### 2.7 Determination of phenolic compounds

The extraction and identification of phenolic compounds were conducted according to our previous method (Xi, Lu, Qun, & Jiao, 2017). Fifteen milliliters of 80% methanol were added to one gram of frozen powder. After mixing by vortexing, the phenolic compounds were extracted ultrasonically at 60°C for 60 min and then centrifuged at 5,000 r/min for 15 min, after which the supernatant was collected. The extraction was repeated three times, and the supernatants were combined, increased to 50 ml with 80% methanol, and stored for phenolic compound determination.

The phenolics were detected by an HPLC system (Waters e2695, USA) equipped with a Sunfire C18 (4.6 mm × 250 mm, 5 μm, Waters) and were detected at wavelengths of 260, 280, and 320 nm with a 2,998 PDA detector. The phenolic compounds were identified by comparing their retention times with the spectral characteristics and peak times of the standard. Their contents were quantified using the standard curve method and expressed in mg/g FW. The three biological replicates were used for each sample.

### 2.8 Statistical analysis

All data are expressed as the mean ± *SE* of three biological replicates. Statistical analysis was performed using the SPSS v19.0 software (SPSS Inc.). Significant differences among the samples were calculated using Tukey's least significant difference test (LSD) at the 5% level.

## 3 RESULTS

### 3.1 Effect of postharvest citric acid treatment on the basic quality index of peach fruit

**Figure 1:**
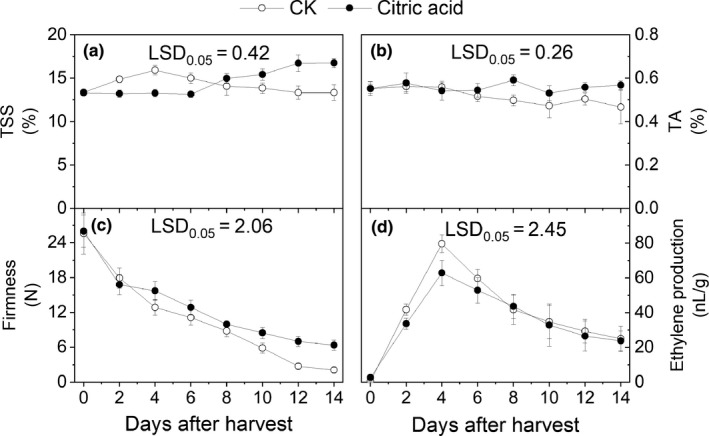
Effects of different treatments on firmness, ethylene production, total soluble solids [TSS], and total acids [TA] in “Hujingmilu” peach fruit during postharvest ripening at 20°C. Error bars are the ±*SE* of the means of three biological replicates. LSD, least significant difference

The TSS contents increased sharply during the first 4 days after harvest in the control group and then decreased to the lowest level at the eighth day after harvest. However, the TSS contents maintained a low level throughout the first 6 days after harvest. In contrast, the TSS levels in the CA group increased rapidly at the sixth day after harvest (Figure 1a), and the contents in the CA group were significantly higher than in control group (*p* < .05). The TA contents decreased in all fruits, but higher TA levels were observed in the CA group compared with the control group during 6 days after harvest (*p* < .05) (Figure 1b). The fruit firmness showed a similar decrease over the storage time (Figure 1c), but the firmness of the CA group was significantly higher than that of the control (*p* < .05). Ethylene production in both control and CA‐treated fruits shared a similar trend (Figure 1d); the climacteric peak occurred at 4 days after harvest, though ethylene release in the CA group was significantly lower than in the control group (*p* < .05).

### 3.2 Effect of postharvest citric acid treatment on the decay incidence of peach fruit

**Figure 2:**
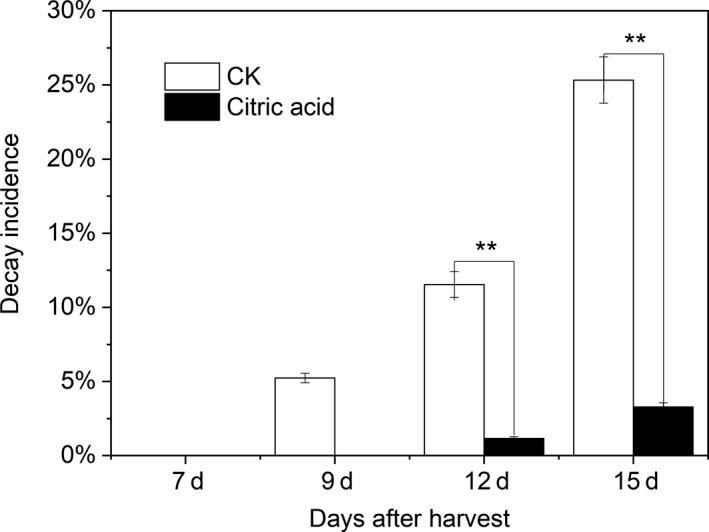
Effects of different treatments on the decay incidence of “Hujingmilu” peach fruit during postharvest ripening at 20°C. Error bars are the ±*SE* of the means of three biological replicates. LSD, least significant difference

Regardless of the control or CA treatment, no fruit decay was observed during the first week after harvest (Figure 2). On the ninth day after harvest, 5% decay incidence was observed in the control, but there were no decayed fruits among the CA fruits. As the storage time was prolonged, the rate of decay increased rapidly. Additionally, 2% decay was also detected in the CA group at 12 days after harvest, which is significantly lower than that in control (*p* < .05). The decay incidence reached 25% in the control during the second week after harvest, but it remained below 4% in the CA group.

### 3.3 Effect of citric acid treatment on the soluble sugars and organic acids in peach fruit

**Figure 3:**
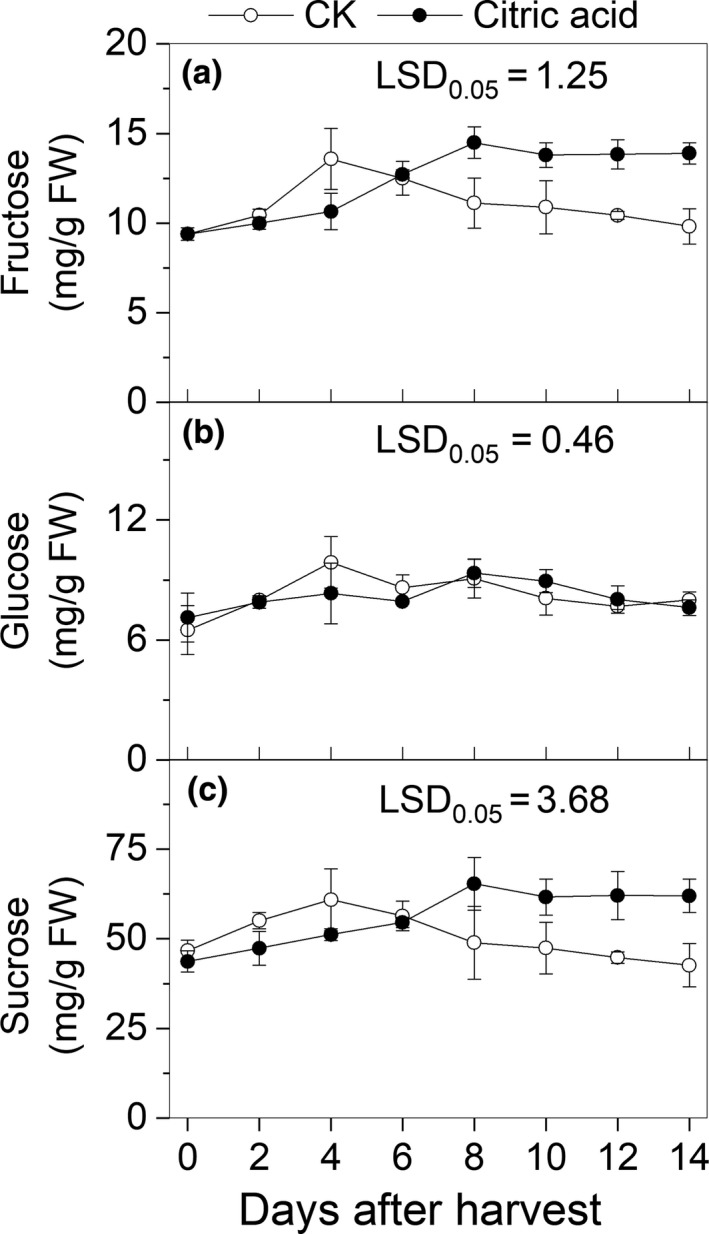
Effects of different treatments on the soluble sugar contents in “Hujingmilu” peach pulp after harvest at 20°C. Error bars are the ±*SE* of the means of three biological replicates. LSD, least significant difference

The main soluble sugars in the fruit were sucrose, fructose, and glucose, and no sorbitol was detected in peach fruit (Figure 3). Sucrose, fructose, and glucose in the control group showed a remarkable increase with climacteric ethylene production, and their contents were significantly lower in the CA treatment fruits than that in the control group during 4 days after harvest, but their contents were significantly higher in the CA group than in the control group (*p* < .05) after the eighth day of harvest.

**Figure 4:**
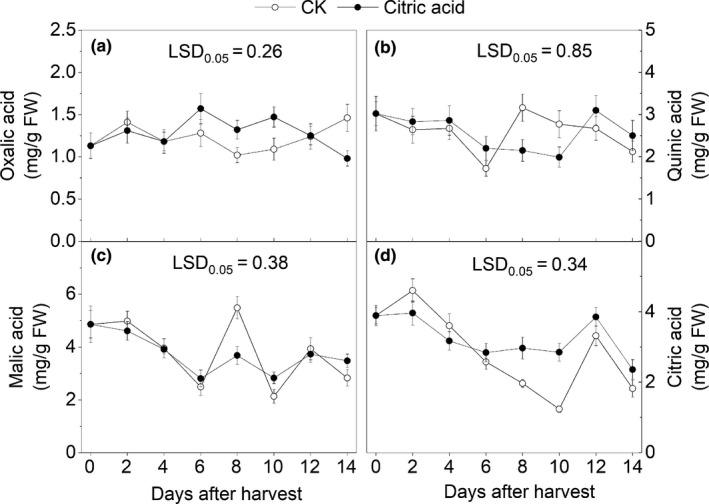
Effects of different treatments on the organic acid contents in “Hujingmilu” peach peel after harvest at 20°C. Error bars are the ±SE of the means of three biological replicates. LSD, least significant difference

Oxalic acid, quinic acid, malic acid, and citric acid are organic acids detected in "Hujingmilu" peach fruit (Figure 4). Among them, malic acid and citric acid are the main organic acids in the fruit. All detected organic acids showed a decreasing tendency at first, after which they increased during the storage period. There was no significant difference in the malic acid contents between the CA and control groups during the early stage of storage (*p* < .05). On the eighth day of harvest, the malic acid contents in the control group were significantly higher than those in the CA group (*p* < .05). During the late storage period, the malic acid contents in the CA treatment group maintained a relatively stable level. During the storage process, the citric acid contents in CA‐treated fruit were substantially greater than the control group from the eighth day (*p* < .05).

### 3.4 Effect of postharvest citric acid treatment on volatile compounds in peach fruit

**Figure 5:**
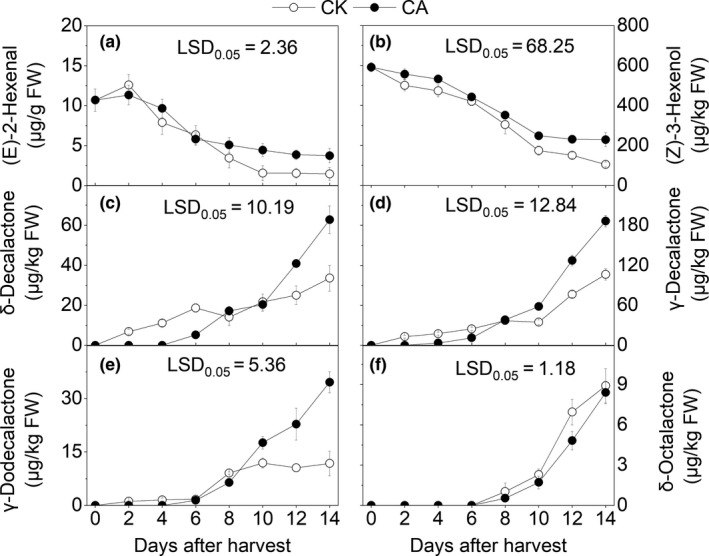
Effects of different treatments on the aroma production from “Hujingmilu” peach fruits peel during postharvest at 20°C. Error bars are the ±*SE* of the means of three biological replicates. LSD, least significant difference

(*Z*)‐3‐hexenol and (*E*)‐2‐hexenal were detected as volatiles with green odor, and their contents decreased sharply during the storage period (Figure 5a and b). No significant difference was observed in (*Z*)‐3‐hexenol and (*E*)‐2‐hexenal between the CA and control groups during the first week after harvest, though remarkably higher contents of (*Z*)‐3‐hexenol and (*E*)‐2‐hexenal were observed in the CA group compared with the control during the second week after harvest (*p* < .05).

Four lactones with fruity and sweet odor volatiles were identified from the tested peaches, including δ‐octalactone, δ‐decalactone, γ‐decalactone, and γ‐dodecalactone (Figure 5c–f). The levels of the four lactones were low during the early harvesting period, though their contents increased rapidly at the eighth day after harvest. No significant differences were observed in the levels of the four lactones between the CA and control groups during the first 6 days after harvest, though the contents of δ‐decalactone, γ‐decalactone, and γ‐dodecalactone were significantly higher in the CA group than those in the control fruits after 8 days of harvest (*p* < .05).

### 3.5 Effect of postharvest citric acid treatment on the polyphenol contents in peach fruit

**Figure 6:**
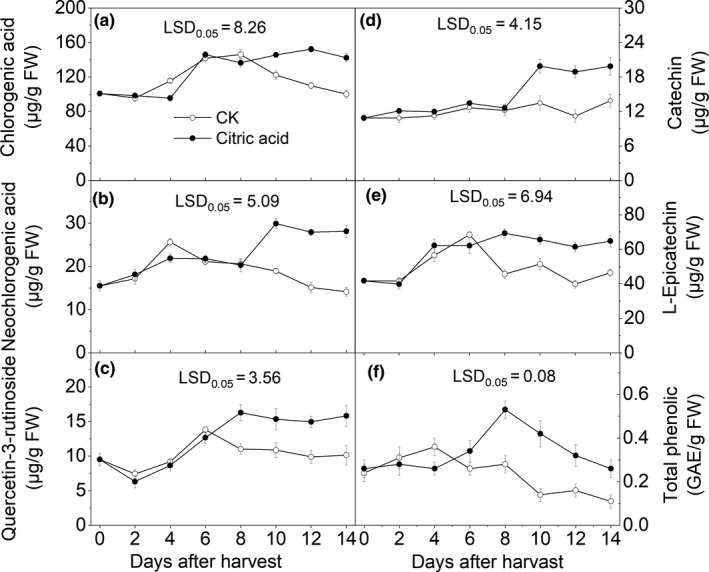
Effects of different treatments on the individual phenolic contents of “Hujingmilu” peach fruits during postharvest at 20°C. Error bars are the ±*SE* of the means of three biological replicates. LSD, least significant difference

The phenolic substances detected from “Hujingmilu” peach fruit mainly included chlorogenic acid, neochlorogenic acid, catechin, L‐epicatechin, and quercetin‐3‐rutinoside (Figure 6). Chlorogenic acid and L‐epicatechin were the predominant phenolics in the tested peaches. Though no significant difference in the contents of the identified phenolics was detected between the CA group and the control fruits during 6 days after harvest, significantly higher catechin and L‐epicatechin contents were maintained in the CA group compared with the controls on the eighth day after harvest (*p* < .05).

## 4 DISCUSSION

Peach fruit is typical climacteric fruit and experiences fast ripening after harvest, which makes it easily perishable and have a limited shelf life (Farooq et al., 2018). Exogenous organic acid treatment has been reported to have potential to maintain postharvest quality in fruit (Huang, Jian, Jiang, Duan, & Qu, 2016) and plays a role in regulating plant maturation and senescence (Choehom, Ketsa, & van Doorn, 2004; Darandeh & Hadavi, 2012). Citric acid, as an important organic acid in plants, can also efficiently improve postharvest fruit quality, which has been proven in studies on apple and litchi (Jiang, Fu, Zauberman, & Fuchs, 1999; Santerre, Cash, & Vannorman, 1988). The quality of harvested peach fruit is generally indicated by its TSS, TA, firmness, ethylene release, decay incidence, and several key phytochemical‐related flavor and nutrition, including organic acid content, soluble sugar content, aroma volatiles, and phenolics content. To the best of our knowledge, this is the first investigation focused on the effects of citric acid on texture, taste, aroma, and nutrition quality of postharvest peach fruit during shelf life conditions.

In general, the cell wall mesocarp disassembly occurs during fruit ripening, meaning that the fruit will rapidly soften and show a sharp loss in firmness (Brummell, 2006), which promotes postharvest decay and shortens the shelf life of fruits. Even the firmness of cherimoya treated with citric acid decreased, but at a significantly slower rate was observed in CA‐treated fruits than among the controls, which mainly because citric acid treatment retarded or suppressed the expression of *AcXETs*, *AcEXPs,* and *AcPE* (Liu et al., 2016). Cassava starch edible coatings and citric acid dipping promoted a decrease in the respiration rate of mango slices, with values up to 41% lower than the control fruit. This treatment also promoted better preservation of texture and color characteristics of mangoes and delayed carotenoid formation and browning reactions during mango storage (Chiumarelli, Pereira, Ferrari, Sarantópoulos, & Hubinger, 2010). Chitosan citric acid treatment increased the shelf life of mangoes by at least 3 weeks and maintained the red coloration of litchi fruit pericarp by increasing activities of POD (Ducamp‐Collin, Ramarson, Lebrun, Self, & Reynes, 2008). In the present study, we found that CA treatment remarkably slowed down peach fruit softening throughout the storage period at shelf life temperature, with an evident difference being observed from the second day of storage. TSS and TA are the crucial quality indexes of ripening in peach fruit. In the present study, our CA treatment maintained a slow ripening process after harvest, which tends to decrease TSS and increase TA; similar results were also found in cherimoya (Liu et al., 2016).

The variation in firmness, TSS, and TA are closely related to ethylene production (Defilippi, Kader, & Dandekar, 2005; Moya‐Leon, Vergara, Bravo, Montes, & Moggia, 2006). Inhibiting fruit ethylene synthesis and reducing fruit respiration intensity are effective means to prolong fruit storage time. In fact, citric acid can participate in the tricarboxylic acid cycle (TCA) during fruit ripening and senescence. When exogenous citric acid enters the cycle, it directly causes feedback inhibition of citrate synthase and accelerates the progression of the TCA, thus reducing the amount of ethylene produced and delaying the peak ethylene release (Hussain et al., 2017). Previous studies have shown that citric acid can inhibit fruit respiration (Chalmers & Faragher, 1977); increase POD, CAT, and SOD activity; and reduce O^2‐^ and H_2_O_2_ contents in pear fruit (Robards, Prenzler, Tucker, Swatsitang, & Glover, 1999). In this study, we found that the citric acid treatment of postharvest fruits reduced the peak value of ethylene production in peach fruit, resulting in the effective inhibition of TSS synthesis in the early stages of storage and retarding the softening and decline of TA in peach fruit during storage. At the same time, our CA treatment prevents significantly the fruit decay of peaches. In citrus, citric acid inhibits production of H_2_O_2_ in peel disks and enhances the pathogenicity of both *Penicillium digitatum* and *P. expansum* (Macarisin et al., 2007), which suggested that CA treatment can prevent decay incidence of fruits by improving their resistance ability to microbiological diseases.

Sugar and organic acids constitute the background flavor of the fruit. This study found that the soluble sugar contents in peach fruit increased in the early stages of storage. However, with the prolongation of the storage time, this began to decrease, which is in accordance with a previous study (Borsani et al., 2009). Citric acid treatment delayed the increase in glucose, fructose, and sucrose contents during the early postharvest storage period, which was consistent with the downward trend of citric acid treatment in slowing the production of soluble solids. As the peach fruit ripens and undergoes senescence, the organic acids gradually degrade; in contrast, delaying organic acid degradation can maintain fruit flavor quality (Fan, Argenta, & Mattheis, 2002). Moreover, exogenous citric acid provides more precursors for ATP production, ensuring a sufficient intracellular ATP supply, while continued extracellular ATP signaling attenuates stresses, delays senescence, and maintains fruit quality (Aghdam, Jannatizadeh, Luo, & Paliyath, 2018). During postharvest storage, as hydration continues to increase, citric acid is continuously consumed as a respiratory matrix through glycolysis, tricarboxylic acid cycle, and gluconeogenesis. We found that citric acid treatment promoted the increase in organic acid contents in the fruit during the late storage period, which correspondingly increased the ATP supply to prevent senescence or resist postharvest stress. Aroma substances play a decisive role in the formation of fruit flavor quality. Citric acid treatment delayed the decrease in the green odor C6 aroma volatiles and the increase in characteristic lactones with fruity odors in the early stages of storage, which prompted the normal conversion of green to fruity odors during peach fruit ripening, maintaining the aroma quality of postharvest peaches.

The polyphenolic compounds are the most important functional component and are also important indicators of nutritional quality (Saidani et al., 2017). Many studies have found that phenolic compound metabolism is relatively stable, and its content slowly decreases during storage. Phenylalanine ammonia‐lyase is a key enzyme for phenolics biosynthesis in the phenylpropanol pathway. In apples, the amount of ethylene released from fruits is related to the accumulation of phenylalanine ammonia‐lyase and phenolics (Penniston, Nakada, Holmes, & Assimos, 2008). The metabolism of phenylpropanol is one biosynthetic pathway that produces secondary metabolites such as phenols. The above results suggested that citric acid may regulate the metabolic synthesis of phenolic compounds by inhibiting the release of ethylene, thereby affecting the contents of phenolic compounds. Additionally, the phenolic compounds in fruits have antioxidant properties to prevent the collapse of postharvest fruit tissues (Dambolena et al., 2008). In this study, CA treatment maintained the postharvest quality of peaches by prompting rich phenolics accumulation to resist senescence and stress.

## 5 CONCLUSION

The application of citric acid could reduce decay in postharvest peaches; prolong the texture, taste, aroma, and nutrition quality by maintaining good firmness and high levels of sucrose and fructose; delay large losses of malic acid and citric acid; and remarkably increase the contents of C6 compounds and δ‐decalactone, γ‐decalactone, and γ‐dodecalactone, thus promoting the normal conversion of green odors to fruity odors, and even maintain the rich chlorogenic acid and L‐epicatechin contents. Our findings suggest that treatment with 10 g/L citric acid can provide an effective approach to maintain the postharvest quality and extend the shelf life of peaches.

## CONFLICT OF INTEREST

We declare that they do not have any conflict of interest.

## ETHICAL APPROVAL

This study does not involve any human or animal testing.

## CONSENT FOR PUBLICATION

Not applicable.

## INFORMED CONSENT

Written informed consent was obtained from all study participants.
